# Social and economic predictors of under-five stunting in Mexico: a comprehensive approach through the XGB model

**DOI:** 10.7189/jogh.15.04065

**Published:** 2025-03-14

**Authors:** Brian Fogarty, Angélica García-Martínez, Nitesh V Chawla, Edson Serván-Mori

**Affiliations:** 1 Lucy Family Institute for Data & Society, University of Notre Dame, South Bend, Indiana, USA; 2 Centre for Health Systems Research, the National Institute of Public Health, Cuernavaca, Morelos, Mexico

## Abstract

**Background:**

The multifaceted issue of childhood stunting in low- and middle-income countries has a profound and enduring impact on children's well-being, cognitive development, and future earning potential. Childhood stunting arises from a complex interplay of genetic, environmental, and socio-cultural factors. It requires a comprehensive approach across nutrition, education, healthcare, and poverty reduction sectors to mitigate its prevalence and short- and long-term effects. The Mexican case presents a distinct challenge, as the country has experienced the recent dissolution of social health security programmes, rising poverty rates, and reduced government expenditures for childhood well-being.

**Methods:**

We propose a machine learning approach to understand the contribution of social and economic determinants to childhood stunting risk in Mexico. Using data from the 2006–2018 population-based Mexican National Health and Nutrition Surveys, six different machine learning classification algorithms were used to model and identify the most important predictors of childhood stunting.

**Findings:**

Among the six classification algorithms tested, Extreme Gradient Boosting (XGB) obtained the highest Youden Index value, effectively balancing the correct classification of children with and without stunting. In the XGB model, the most important predictor for classifying childhood stunting is the household's socioeconomic status, followed by the state of residence, the child’s age, indigenous population status, the household’s portion of children under five years old, and the local area’s deprivation level.

**Conclusions:**

This paper contributes to understanding the structural determinants of stunting in children, emphasising the importance of implementing tailored interventions and policies, especially given our findings that highlight indigenous status and local deprivation as key predictors. In the context of diminishing health initiatives, this underscores the urgent need for specific, targeted, and sustainable actions to prevent and address a potential rise in stunting in similar settings.

**Keywords:**

social and economic deprivation, stunting, children, machine learning, XGB model, Mexico

Over the past few years, hard-won progress in reducing global malnutrition has been stymied by the direct and indirect impacts of the COVID-19 pandemic and conflict in Ukraine [1,2]. These impacts include shifting public health priorities, reducing and eliminating nutrition programmes, and disrupting the global supply chain. Childhood malnutrition is particularly insidious due to the short- and long-term effects on the development of individual children, as well as its adverse effects on local communities, regional populations, and entire societies [3,4]. Despite numerous efforts and interventions to tackle the underlying factors associated with childhood malnutrition, it remains a persistent and chronic global public health challenge [5]. The scale of the problem is alarming, with international organisations reporting 148.1 million children are affected by stunting, 45 million by wasting, and 37 million by being overweight [6].

Researchers seeking a better understanding of childhood malnutrition to guide the development of targeted interventions have been hindered by the limited availability of high-quality and harmonised data. This challenge is especially pronounced when studying vulnerable groups, such as indigenous populations in low and middle-income countries (LMICs) [2]. Given the available data, a critical question is whether we can leverage Artificial Intelligence (AI) to enhance our insight into the determinants of childhood malnutrition and design new approaches for mitigating its prevalence and impact [7]. In this paper, we employ machine learning (ML) classification algorithms, a subset of AI, to assess the influence of social health protection programmes and social and economic factors on the risk of childhood stunting in Mexico between 2006–2018.

Mexico, the world's fifteenth largest economy [8], the tenth most populous country globally and second in Latin America and the Caribbean [9], and highly economically unequal [10], is a compelling case study among LMICs for investigating the multifaceted factors contributing to childhood stunting at the individual, family, and community levels [4]. Mexico's social health system has recently scaled back pivotal health and nutrition initiatives designed to safeguard vulnerable populations, including for indigenous and children under five years old in rural areas [11]. The reduction in government support has imposed additional financial burden on families, resulting in many children foregoing preventative and essential medical care [12]. By not addressing stunting and poor health in childhood, countries like Mexico are creating conditions where a larger proportion of the most vulnerable population are at higher risk of developing chronic health issues in adulthood [13–16]. This perpetuates limited opportunities for social and economic advancement within rural communities, further exacerbating inequities between them and their urban peers [11,14,17,18].

The intricate interdependence of health outcomes with other social subsystems (*e.g.* education, economic, environmental, *etc.*) [19,20] calls for a comprehensive analytical approach to the problem of child malnutrition [21]. Prior research has recognised the potential of robust predictive models to inform the design of innovative strategies aimed at effectively addressing the factors that influence the prevalence and persistence of child malnutrition [22,23]. However, the paucity of high-quality, longitudinal data has limited the implementation of predictive models in this area [2].

In lieu of high-quality nutrition data, particularly in LMICs, researchers have increasingly adopted ML as an alternative modelling approach to applied statistical analysis [24]. For example, ML has recently been used to study childhood malnutrition in Bangladesh [25–27], Pakistan [28], Philippines [29] and Rwanda [30]. Machine learning is adept at sifting through large and complex data sets to discern patterns and identify latent factors involved in nutrition-related outcomes and public health [31]. Other recent applications in public health include creating predictive models for nutritional risk assessment, regional health needs, disease patterns, and healthcare expenditure forecasts [32].

## METHODS

We conducted a pooled cross-sectional analysis using data from three National Health and Nutrition Surveys (Encuesta Nacional de Salud y Nutrición, ENSANUT) carried out in 2006, 2011/12, and 2018/19. Each survey collected social, demographic, and health-related data from the Mexican population and recorded information on the coverage of health services. The methodological details of the surveys have been described elsewhere [33,34]. However, briefly, ENSANUT is a probabilistic, population-based, stratified, two-stage, cluster-design survey conducted in all 32 Mexican states, with samples representative at national and state levels and disaggregated into urban and rural strata. Data are gathered during home visits through electronically captured face-to-face interviews after respondents provide informed consent or assent. The protocols for the three surveys were approved by the Research Ethics and Biosecurity Committees of the authors’ institution in Mexico. Data collected are available to the public and can be downloaded from https://ensanut.insp.mx/.

We analysed the survey modules on sociodemographic and household characteristics, as well as the module on anthropometric measurements. After combining the data from the three survey rounds, we selected only those children with complete information for all relevant characteristics. Consequently, from the initial sample of 21 304 children, we excluded 501 due to missing data, resulting in a 2.4% reduction in sample size. We then compared the children included in the final analysis (n = 20 803) with those excluded, across various relevant characteristics, and found no significant differences between the two groups.

### Outcome variable

We assessed the nutritional status of the children according to the World Health Organization (WHO) child-growth standards based on height and age [35,36]. We defined a binary variable to classify children as stunted (1) if their height-for-age Z-score was <−2 and not stunted (0) if it was ≥ −2. All observations were within the interval from − 6.0 to + 6.0 Z-score (for length or height for age) for identifying valid observations.

### Social and economic predictors

We selected demographic and socioeconomic predictors for our ML models based on prior literature, theoretical expectations, and bivariate statistical analyses [17,37–39]. Since we had categorical and numeric predictors, two types of bivariate statistical analysis were conducted. First, logit regression analyses were used with stunting as the outcome variable and each predictor was tested individually. Second, χ^2^ analyses were performed between stunting and each predictor, where quartile versions of the numeric predictors (*i.e.* categorical versions were created) were used. Predictors that did not have a statistically significant relationship with stunting in the logit regression and the χ^2^ analysis were excluded from the final set of variables used in the ML models. This process only excluded two variables – an indicator variable for whether the head of household was working and the proportion of the household 65 years old or older. To reduce potential collinearity, we removed predictors that were highly correlated and measured the same concept, keeping only the most relevant ones based on substantive importance. Specifically, we excluded adult equivalency and retained household size. Additionally, we removed predictors for the proportion of household members aged 15–64 years and over 65 years and kept the predictors for the proportion of household members under five years and under 15 years. Further, in our ML models, we use the non-categorical versions of the numeric predictors to preserve variation in our predictors’ values. We tested an all-categorical version of the predictors, and the classification metrics were the same or worse depending on the algorithm.

Three groups of predictors were selected:

1. Individual: age (years) and sex (male = 1, female = 0) of child

2. Household: age, sex, education level, and marital status (free union/married, separated/divorced/widowed, single) of household’s head, the type of health insurance (social security, the extinct Seguro Popular de Salud, private, mixed, other or none), the household’s size, the proportion of household members aged 0–5 years and under 15 years, indigenous status (defined according to the official Mexican definition: those residing in a household where the head of the family, a spouse and/or an older relative such as a grandmother speaks an indigenous language) [40,41], participating in the Mexico’s Conditional Cash Transfer Progresa/Prospera/Oportunidades (CCT-POP) programme [42–44], and a factorial index of assets and housing materials as a measure of socioeconomic status (SES) [45–54]. We calculated the index based on the factor loadings for the 2006 survey. This index consolidates a broad range of household characteristics into a single variable. These characteristics include asset ownership (television sets, radios or stereos, iron, refrigerator, gas cooker, blender, washing machine, telephone, car or motorised transport), housing conditions (roofs, walls, and floors made of durable materials, number of bedrooms), and access to essential public services (electricity, running water and sewage services). To ensure consistency and comparability across different survey years, we applied the factor weights estimated from the 2006 survey (the first round of data collection) when constructing the index. The range of the SES index was from −3.997 to 2.069; more positive scores indicate higher SES, while lower SES households have more negative scores.

3. Place of residence: we also included state and a social deprivation index for the place of residence based on municipal access to basic public services, housing conditions, and salary [55]. This index ranged from −2.982 to 3.652, with higher values indicating greater social and economic development in the municipality.

**Table 1** provides descriptive information about the variables used in our analysis. Of note, the overall percentage of children classified as stunted was 13.9% in our analytical sample. Over the three waves, stunting increased from 13.3% in 2006 to 15.3% in 2018.

**Table 1:** Sociodemographic profile of sample

Sample size (n) = 20 803	Mean or % (95% CI)
Child	
*Stunted*	13.86% (13.40, 14.34)
*Stunted in 2006 wave*	13.28% (13.40, 14.34)
*Stunted in 2012 wave*	13.76% (13.07, 14.48)
*Stunted in 2018 wave*	15.27% (14.16, 16.45)
Age, years	2.67 (2.65, 2.69)
Female	49.19% (48.51, 49.88)
Household head	
*Age, years*	40.23 (40.05, 40.42)
*Schooling, years*	7.45 (7.38, 7.50)
*Indigenous*	12.50% (12.06, 12.96)
*Male*	80.44% (79.89, 80.98)
Marital status	
*Married*	86.45% (85.97, 86.91)
*Separate/divorced/widowed*	10.92% (10.50, 11.35)
*Never married*	2.63% (2.42, 2.86)
Household	
*Household size*	5.24 (5.21, 5.27)
*Proportion of children 0–5 y*	28.7 (28.5, 28.8)
*Proportion of children <15 y*	44.2 (44.0, 44.4)
*Socioeconomic status*	−0.045 (−0.058, 0.031)
*Social deprivation index*	−0.602 (−0.616, 0.589)
Participating in a conditional cash transfer programme	30.64% (30.01, 31.27)
Health insurance	
*Social security*	27.39% (26.79-28.00)
*Seguro popular*	38.55% (37.88-39.21)
*Private/mixed/other*	1.48% (1.32-1.66)
*None*	32.58% (31.95-33.23)
State of residence	
*Aguascalientes*	3.88% (3.62, 4.15)
*Baja California*	2.37% (2.17, 2.59)
*Baja California Sur*	2.60% (2.39, 2.82)
*Campeche*	3.20% (2.96, 3.45)
*Coahuila*	2.59% (2.38, 2.82)
*Colima*	2.62% (2.41, 2.85)
*Chiapas*	4.11% (3.84, 4.39)
*Chihuahua*	2.72% (2.50, 2.95)
*Ciudad de México*	2.06% (1.88, 2.27)
*Durango*	3.38% (3.14, 3.64)
*Guanajuato*	3.39% (3.15, 3.65)
*Guerrero*	3.71% (3.46, 3.98)
*Hidalgo*	3.19% (2.95, 3.44)
*Jalisco*	3.23% (3.00, 3.48)
*Estado de México*	3.21% (2.98, 3.46)
*Michoacán*	3.27% (3.03, 3.52)
*Morelos*	3.18% (2.95, 3.43)
*Nayarit*	2.85% (2.62, 3.08)
*Nuevo León*	3.09% (2.86, 3.33)
*Oaxaca*	4.05% (3.79, 4.33)
*Puebla*	3.25% (3.01, 3.50)
*Querétaro*	2.96% (2.73, 3.20)
*Quintana Roo*	2.96% (2.74, 3.20)
*San Luis Potosí*	2.93% (2.70, 3.17)
*Sinaloa*	2.60% (2.39, 2.82)
*Sonora*	2.59% (2.38, 2.81)
*Tabasco*	4.03% (3.77, 4.31)
*Tamaulipas*	2.86% (2.64, 3.10)
*Tlaxcala*	3.69% (3.44, 3.96)
*Veracruz*	3.21% (2.98, 3.46)
*Yucatán*	3.11% (2.88, 3.35)
*Zacatecas*	3.13% (2.90, 3.38)

CI – confidence interval

### Machine learning approach

Prior to specifying and evaluating classification algorithms, we first pre-processed our data. To maximise the number of observations in our models, we imputed missing values in numeric predictors (using the mean) and in categorical predictors (using the mode). No numeric predictor had an absolute difference between the mean and median greater than 1.5 times the interquartile range. Therefore, missing values were imputed using the mean rather than the median. Since the amount of missing data was small (only five predictors had missing values, consisting of a total of 709 observations) and did not appear to be influenced by or related to other predictors, this simple imputation approach performed similarly to more complex imputation methods (*e.g.* K-Nearest Neighbors imputation). We next dummy encoded all of the categorical predictors. All numeric predictors were normalised as is required for some algorithms we examined (*e.g.* neural networks) and as a general practice to improve learning efficiency [56]. We also applied a zero-variance filter to remove any predictors that consisted of a single value. However, none of the predictors were found to have zero variance. Additionally, we specified a mixed generalised linear model encoding for households’ states instead of a fixed effect specification. Analysis was conducted using *R,* version 4.2.2 (Vienna, Austria) with the ‘tidymodel’s’ framework and packages [57].

We split the data into a training set (75% of observations) and a testing set (25% of observations). Due to the imbalanced nature of our outcome variable, stunting, we used simple up-sampling of the minority class to create even classes in the training data. Simple up-sampling performed better than other techniques such as Synthetic Minority Over-sampling Technique SMOTE [58]. Following standard guidance, the up-sampling was only applied in the training data and not the testing data [57]. If we did not apply oversampling (or under sampling) in this case, the classification algorithms would likely overfit the ‘not stunted’ outcome (the majority class). As a result, commonly used evaluation metrics, including accuracy and Receiver Operating Characteristic Area Under the Curve (ROC AUC), would indicate better performance than what was achieved. For example, if a model classified all children as not stunted and none as stunted, the accuracy would be 0.861 (the proportion of children in the data who are not stunted).

Since we had no a priori expectation about the best model for predicting childhood stunting, we initially tested 10 commonly used algorithms for binary classification. Four algorithms performed poorly, based on classification metrics, and were excluded from further consideration. The excluded classification algorithms were Support Vector Machines (Radial Basis Function specification), K-Nearest Neighbors, Classification and Regression Trees (CART), and Bagged CART [57]. Six algorithms performed similarly in initial testing analysis: logistic, random forest (RF), neural network (NN), multivariate adaptive regression splines (MARS), Extreme Gradient Boosting (XGB), and Light Gradient Boosting (LGB).

In the training phase, each algorithm’s parameters were tuned over a grid (consisting of 25 different parameter values) and performance was evaluated using stratified 10-fold cross-validation resampling. Due to the outcome variable’s imbalanced classes, we used the Youden Index (YI) to determine each algorithm’s optimal specification. Youden Index takes into account both sensitivity and specificity (sensitivity + specificity −1) and is insensitive to the distribution of observations across a variable’s categories [59,60]. Maximising this metric ensures that each algorithm’s specification was optimised for correctly classifying both children who are stunted and not stunted. Using common performance metrics such as ROC AUC and accuracy for this purpose can yield misleading results when working with imbalanced data [57,61]. The optimal parameter specifications, based on the training data, were then selected for each classification algorithm in the testing data. See **Box 1** for the algorithms’ tuned specifications.

Box 1 **Classification algorithms’ final tuning specifications**

● LGB: tree depth = 4, learning rate = 0.000985, minimum loss reduction = 1.72e-10, minimal node size = 34, number of randomly selected predictors = 10, number of trees = 1661

● Logistic: amount of regularisation = 0.0549, proportion of Lasso penalty = 0.0668

● MARS: degree of interaction = 1

● NN: number of hidden units = 1, amount of regularisation = 1.50e-10, number of epochs = 694

● RF: number of randomly selected predictors = 1, minimal node size = 16, number of trees = 265

● XGB: tree depth = 2, learning rate = 0.0239, minimum loss reduction = 11.2, minimal node size = 22, proportion of observations samples = 0.692, number of trees = 935

## RESULTS

In **Table 2**, we present performance metrics for the six classification algorithms using the testing data. Given the imbalanced classes of the outcome variable, we use YI for determining the highest performing algorithm. Additional metrics, which are commonly used in classification, are provided in **Table 2** for transparency. Although the algorithms performed somewhat similarly, XGB had the highest YI value (YI = 0.255). XGB also had the highest Matthew’s Correlation Coefficient (MCC) value, which is another commonly used metric for evaluating classification performance in the presence of imbalanced data. This means that XGB achieved the highest performance, effectively balancing the correct classification of children with stunting (*i.e.* sensitivity) and without stunting (*i.e.* specificity). Random Forest was the best algorithm for classifying children without stunting (specificity = 0.779) but performed poorly at classifying children with stunting (sensitivity = 0.454). Even though we used oversampling in training, the fact that RF had the highest accuracy (accuracy = 0.734) in testing highlights the issue of relying on performance metrics that are sensitive to imbalanced data.

**Table 2:** Performance metrics

Variables	LGB	Logistic	NN	MARS	RF	XGB
YI	0.248	0.236	0.241	0.235	0.233	0.255*
ROC AUC	0.664	0.674*	0.666	0.670	0.669	0.672
MCC	0.181	0.172	0.169	0.168	0.185*	0.185*
						
Specificity	0.697	0.688	0.624	0.661	0.779*	0.692
Sensitivity	0.551	0.548	0.617*	0.574	0.454	0.563
Accuracy	0.677	0.669	0.623	0.649	0.734*	0.674

LGB – Light Gradient Boosting, MARS – Multivariate Adaptive Regression Splines, MCC – Matthew’s Correlation Coefficient, NN – Neural Network, RF – Random Forest, ROC AUC – Receiver Operating Characteristic Area Under the Curve, XGB – Extreme Gradient Boosting, YI – Youden Index

*Denotes the classification algorithm(s) with the highest value for each performance metric. If multiple algorithms achieve the same highest value for a metric.

**Figure 1:**
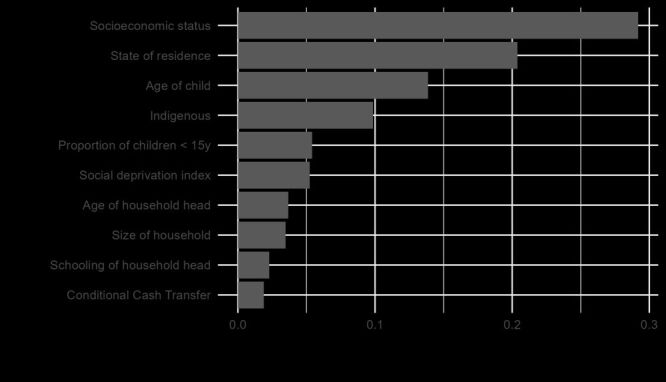
Extreme Gradient Boosting (XGB) variable importance.

**Figure 1** illustrates the 10 most important predictors contributing to XGB’s classification performance. The most important predictor is SES (socioeconomic status of the household), followed by State (pooled effect of Mexican states), child’s age, indigenous status, number of children <15 (household’s portion of children under 15 years old), deprivation index (local area’s deprivation level), age of household head (HH Age), household size (number of members), schooling of household head (HH Edu), and CCT-POP programme.

Lower in importance than one might expect is household participation in Mexico’s CCT-POP programme. This programme was designed to improve the health and well-being of Mexicans, especially children. The CCT programme, technically part of Oportunidades/Prospera, initially focused on conditional cash transfers to low-income families for poverty alleviation but subsequently expanded to encompass broader social protection measures addressing various well-being dimensions [11]. Mexico also implemented child nutrition programmes, targeting marginalised communities with initiatives such as supplementary feeding and nutrition education [62]. Programme effectiveness varied and was contingent on factors like design, funding, and evolving social and economic contexts.

## DISCUSSION

Utilising a ML approach, this paper underscores the significance of family socioeconomic status, community deprivation levels, state of residence, and indigenous status in understanding childhood malnutrition in Mexico. These structural factors collectively highlight the profound impact of poverty on childhood stunting, with potential short- and long-term consequences not only for individual children and families but also for local and regional communities. Addressing the influence of these factors on childhood stunting, and consequently tackling poverty itself, necessitates synergistic partnerships between governmental and non-governmental organisations (NGOs). Such collaborations can harness the strengths of governmental bodies (*e.g.* resources, infrastructure, policy management) and non-governmental entities (*e.g.* flexibility, technical expertise, community engagement strategies) to deliver coordinated and sustainable interventions aimed at mitigating persistent public health issues such as childhood malnutrition.

Our research leverages ML to transcend traditional analyses, offering a comprehensive perspective on the social and economic predictors of stunting, which have been underexplored in the Mexican context. It is crucial to note that our study’s aim is not to compare different ML algorithms to identify the highest performer for modelling childhood stunting. Rather, it aims to illustrate how ML can complement existing research by revealing nuanced insights into the structural determinants of stunting that may escape traditional statistical models. This approach allows for a more robust understanding of the factors driving childhood stunting in Mexico and similar contexts [30,63,64].

While our research indicates that the CCT-POP programme in Mexico may have had a limited influence on addressing childhood stunting, the programme initially succeeded in narrowing inequality gaps and reducing chronic malnutrition in Mexico when launched in 1999 [65–67]. Our study pools survey data from the 2006, 2012, and 2018 ENSANUTs and thus we are capturing the programme’s importance from 2006–2018. During this period, the programme became increasingly ineffective and unsustainable due to growing resource constraints, shifting priorities towards other policy initiatives, and waning political support across multiple presidential administrations [42,68]. Because politicians typically gain credit only for initiating new government programmes, incoming administrations frequently phase out or reduce funding for existing programmes that lack widespread public support [69]. Social welfare programmes can be especially susceptible to diminished investment or complete elimination under new administrations as they often only benefit a narrow segment of the population [70]. Curtailing or eliminating these programmes disproportionality impacts a society’s most vulnerable groups (*e.g.* the indigenous population in Mexico) and exacerbates existing health inequalities [69].

This raises the critical question of how to foster public-private partnerships that can develop effective and sustainable health, nutrition, and education programmes, especially tailored to the needs of young children, and are resistant to political changes. Private donors and NGOs in these partnerships may hesitate to continue funding programmes with limited impact on those most in need, suffer from wasteful management, and lack long-term public commitment. While studies like ours help identify factors that affect public health issues (*e.g.* childhood malnutrition), the effectiveness of interventions is contingent on the implementation, management, and commitment of governmental and NGOs [71].

Our study also identifies predictors previously highlighted in research as significant determinants of childhood stunting. These include child age [64,72], the presence of young children in households [73], and the age and level of education of the household head [4,74]. Designing programmes to address these determinants can be relatively straightforward and require low-cost interventions [68]. For example, if the risk of stunting monotonically decreases for a child overtime, then nutrition and health interventions can be targeted to the ages that generate the most beneficial effect [3]. In contrast, tackling the influence of structural factors in childhood stunting poses a substantially more challenging problem for researchers, policymakers, and stakeholders [3,4].

This study’s use of ML was motivated by the goal of identifying overlooked determinants of childhood stunting. In contrast to the deductive research process of testing theory-derived hypotheses using statistical inference, the inductive and conceptual-based approach of ML explores and leverages the data to identify patterns and relationships that may have eluded or been discarded by prior research. Through its application, we identified the importance of structural factors impacting childhood stunting and raised new questions about the role of Mexican states and social security programmes in childhood stunting and malnutrition more broadly. Machine Learning’s rigorous inductive approach to quantitative data analysis provides researchers innovative procedures to generate new insights and refine hypotheses and theories about a topic [75]. This approach is particularly useful in public health research, where many complex factors can contribute to health outcomes [76]. Critically, ML is not meant to replace traditional statistical modelling or qualitative analysis in public health research. Instead, it should be considered an additional methodological tool for public health researchers to use in tandem with traditional methods in developing a comprehensive understanding of various health outcomes [75].

Effectively tackling stunting in indigenous children requires a holistic public health approach. This involves implementing new policies and targeted programmes, revitalising social security initiatives, and consolidating them for real-time tracking of health and nutrition conditions among vulnerable population groups. To secure the well-being of indigenous populations, a collective regional agenda should be devised, integrating stakeholders, governments, research teams, quality data collection, and AI utilisation to anticipate actions, reduce costs, and enhance the social and economic development of these communities.

We demonstrate the impact of poverty at the family and community levels on childhood malnutrition. The results illustrate how the deficiencies of social security programmes in Mexico exacerbated existing inequities between indigenous groups and the broader population [67]. Our findings confirm age [64,72], the presence of more young children in households [73], and the age and education of the household head [4,74] as important predictors of stunting in children under five. Structural predictors highlight the profound impact of poverty at household and community levels, emphasising the potential negative consequences when social security programmes become unsustainable or ineffective [68]. Despite the initial success of CCT-POP programme had positive impacts on child health and nutrition outcomes [77], challenges in sustaining a trans-governmental programme and limited studies on long-term impacts hinder a comprehensive understanding of the chronic problem of malnutrition, particularly in the indigenous population [78]. The individual Mexican states also have an important contribution to stunting in children under five years. More exhaustive research is required to understand the intersection of poverty and social security programmes available at different levels of government and to understand the actual weights of structural determinants of stunting at the community level.

Current evidence shows how persistent socioeconomic disparities significantly impact stunting rates, particularly among vulnerable populations. Stunting in Mexico remains a critical public health issue, with a 16.3% prevalence in 2018, notably higher in rural southern regions and among low SES households [79,80]. Economic inequality restricts access to nutritious food and adequate healthcare, exacerbating chronic malnutrition. Systemic issues such as inadequate maternal health services, poor sanitation, and limited parental education also contribute to the problem [81]. The dismantling of social health programmes since 2018 has further exacerbated stunting rates, particularly following the COVID-19 pandemic. Indigenous households and those with the lowest SES face the greatest risk, underlining the need for targeted health-financial protection measures and addressing structural discrimination in healthcare [82,83].

The negative effect of stunting in health and neurodevelopment in indigenous populations, under a cycle of life perspective, is limited in the literature [84]. Beyond biological adaptations [3,28], concerns and questions revolve around addressing structural factors like inadequate social security programmes and limited resources. This raises the question of how to encourage public and private investment in high-quality, sustainable health, nutrition, and education programmes during the crucial ‘one hundred days’ window. Additionally, there is a consideration of whether and how civil organisations can efficiently facilitate these benefits for those most in need [71]. The multidimensional approach of stunting requires a comprehensive approach to how poverty and racial and ethnic disparities interact under cultural and social norms to understand the relevance of tailored programmes for vulnerable populations like indigenous [4,85]. Although previous studies found that these programs significantly improved children’s nutrition and reduced illness rates and stunting [65,66], the programmes only play a minor role in our results. This underscores the influence of structural factors, such as poverty and deprivation, in understanding childhood stunting in Mexico. We also found that the CCT-POP Programme did not have a statistically significant effect on childhood stunting in separate logit and complementary log-log regressions with fixed and random effects specifications.

Evidence reported in Latin American countries describes similarities in structural factors related to facilities, health care programmes, and vulnerable subgroups, such as children. Crucial points emerge when comparing crop and meat producers like Uruguay and Argentina to countries with high indigenous populations like Guatemala, Peru, and Mexico, where risk factors reveal similarities in social and economic deprivation contexts, illustrating a nutritional transition where all forms of malnutrition are present. For instance, in Uruguay, 5.45% of children under four in the lower-income tertile are stunted compared to 3.44% in the upper tertile. Excess weight is also a significant public health challenge, particularly among more educated mothers, highlighting the need for targeted interventions [86]. In Argentina, 26% of households have unsatisfied basic needs, 23% receive food assistance, and children are particularly affected by anaemia (15.2%), overweight/obesity (9.9%), and stunting (7.4%) [87]. Stunting prevalence rates (PR) in Peru shows significantly lower stunting in children with high socioeconomic status (PR = 0.25) and highly educated mothers (PR = 0.26), but higher stunting in Indigenous children (PR = 1.3), indicating the need for policies addressing socioeconomic and educational disparities [88]. In Guatemala, stunting prevalence among children is 46.7% (95% confidence interval (CI) = 45.0, 48.6), with higher rates among low-income, low-educated, and indigenous populations [89]. Despite none of the previous studies conducted in Latin America using ML as an approach for predicting stunting in children under five years, our results are consistent with the findings reported in those studies.

Integrating ML with traditional analytical approaches is vital for improving public health research. Machine Learning builds on statistical methods to manage complex patterns and large data sets, enhancing insights and data-driven decision-making [90,91]. However, both approaches face challenges including high-class imbalance, insufficient data variability, and confounding variables that can lead to poor model performance and biased results [92].

A key limitation in our study is the absence of comprehensive historical data, particularly maternal and foetal information covering the first five years of life. This data is crucial for understanding phenomena like stunting. Mexico’s shifting cultural and social landscape, coupled with intermittent social security programmes, further complicates the integration of long-term data, limiting the scope of our analysis.

Effectively tackling stunting in indigenous children requires a holistic approach, recognising it as a triple vulnerability. This involves implementing new policies and targeted programmes, revitalising social security initiatives, and consolidating a nutrition and health observatory initiative for tracking in real-time the condition of multi-vulnerable groups of the population. Finally, to secure the well-being of indigenous populations, a collective regional agenda should be devised, integrating stakeholders, governments, research teams, quality data collection, and AI utilisation to anticipate actions, reduce costs, and enhance the social and economic development of original communities.

While our findings are consistent with prior research, including that socioeconomic status and the child’s age play important roles in understanding childhood stunting in Mexico [93], our study also identifies key factors that have been overlooked or found to have null effects using statistical analysis. Unlike previous studies, we observe that children’s indigenous status and the level of deprivation in their local area are important factors in the prevalence of stunting. Hence, through the application of ML approach, we provide new insight into the determinants of childhood stunting that are not apparent with traditional statistical modelling. Mexico’s scaling back of pivotal health and nutrition initiatives, coupled with the absence of child-focused health and nutrition programmes, portends a substantial increase in childhood stunting and malnutrition, particularly among vulnerable populations such as indigenous and under-five children in rural areas. Given the limited investment in infrastructure and the need for skilled human resources in Mexico and other LMICs to address public health issues in specific groups, research like ours is critically needed by public health officials to help design effective interventions, policies, and programmes.

In sum, addressing malnutrition at the municipal level through sustainable actions supported by community engagement, access to healthy foods, and the collaboration of NGOs and international organisations is essential. Multi-sectoral interventions that integrate both nutrition-specific and nutrition-sensitive approaches, involving government, non-government, and private sectors, are key to improving outcomes like stunting. To break the cycle of poverty in LMICs, regional efforts are needed to monitor childhood malnutrition indicators and strengthen social programmes in education and health, particularly in rural and Indigenous communities [4,68]. Leveraging data science and AI to integrate social determinants of health can enable a comprehensive, inclusive approach to addressing stunting in children under five by capturing diverse influences and informing targeted, equitable interventions and policies [94].

## References

[1] The triple burden of malnutrition. Nat Food. 2023;4:925. 10.1038/s43016-023-00886-8 37985699

[2] Victora CG Christian P Vidaletti LP Gatica-Domínguez G Menon P Black RE Revisiting maternal and child undernutrition in low-income and middle-income countries: variable progress towards an unfinished agenda. Lancet. 2021;397:1388–99. 10.1016/S0140-6736(21)00394-9 33691094 PMC7613170

[3] De Sanctis V Soliman A Alaaraj N Ahmed S Alyafei F Hamed N Early and long-term consequences of nutritional stunting: from childhood to adulthood. Acta Biomed. 2021;92:e2021168. 33682846 10.23750/abm.v92i1.11346 PMC7975963

[4] Vaivada T Akseer N Akseer S Somaskandan A Stefopulos M Bhutta ZA Stunting in childhood: an overview of global burden, trends, determinants, and drivers of decline. Am J Clin Nutr. 2020;112:777S–91S. 10.1093/ajcn/nqaa159 32860401 PMC7487433

[5] World Health Organization (WHO). Reducing stunting in children: equity considerations for achieving the global targets 2025. Geneva 27, Switzerland. 2018. Available: https://www.who.int/publications/i/item/9789241513647. Accessed: 15 January 2022.

[6] United Nations International Children’s Emergency Fund (UNICEF). (WHO) WHO, World Bank Group (WB). Levels and trends in child malnutrition. UNICEF / WHO / World Bank Group joint child malnutrition estimates. Key findings of the 2023 edition. New York, NY. 2023. Available: https://data.unicef.org/resources/jme-report-2023/. Accessed: 13 May 2024.

[7] Liang W Tadesse GA Ho D Fei-Fei L Zaharia M Zhang C Advances, challenges and opportunities in creating data for trustworthy AI. Nat Mach Intell. 2022;4:669–77. 10.1038/s42256-022-00516-1

[8] The World Bank. Mexico overview. The World Bank in Mexico. 2023. Available: https://www.worldbank.org/en/country/mexico/overview. Accessed: 3 October 2024.

[9] The World Bank. Data bank. Population, total. 2023. Available: https://data.worldbank.org/indicator/SP.POP.TOTL. Accessed: 21 June 2024.

[10] Esquivel Hernández G. Desigualdad extrema en México. Concentración del poder económico y político. Ciudad de Mexico, Mexico; 2015. Available: https://oxfammexico.org/desigualdad-extrema-en-mexico/. Accessed: 13 July 2022.

[11] Knaul FM Arreola-Ornelas H Touchton M McDonald T Blofield M Avila Burgos L Setbacks in the quest for universal health coverage in Mexico: polarised politics, policy upheaval, and pandemic disruption. Lancet. 2023;402:731–46. 10.1016/S0140-6736(23)00777-8 37562419

[12] González Block MÁ Reyes Morales H Hurtado LC Balandrán A Méndez E Mexico: health system review. Health Syst Transit. 2020;22:1–222. 33527902

[13] Victora CG Adair L Fall C Hallal PC Martorell R Richter L Maternal and child undernutrition: consequences for adult health and human capital. Lancet. 2008;371:340–57. 10.1016/S0140-6736(07)61692-4 18206223 PMC2258311

[14] Leroy JL Frongillo EA Perspective: what does stunting really mean? A critical review of the evidence. Adv Nutr. 2019;10:196–204. 10.1093/advances/nmy101 30801614 PMC6416038

[15] Henriques A Teixeira V Cardoso HF Azevedo A The influence of stunting on obesity in adulthood: results from the EPIPorto cohort. Public Health Nutr. 2018;21:1819–26. 10.1017/S1368980018000460 29540251 PMC10260921

[16] Alves JGB Alves LV Early-life nutrition and adult-life outcomes. J Pediatr (Rio J). 2024;100:S4–S9. 10.1016/j.jped.2023.08.007 37813343 PMC10960187

[17] Serván-Mori E Quezada-Sánchez AD Fuentes-Rivera E Pineda-Antunez C Hernández-Chávez MDC García-Martínez A Proximal determinants of suboptimal early child development during the first three years of life in socially deprived Mexican contexts. PLoS One. 2023;18:e0291300. 10.1371/journal.pone.0291300 37917638 PMC10621868

[18] Batis C Denova-Gutiérrez E Estrada-Velasco BI Rivera J Malnutrition prevalence among children and women of reproductive age in Mexico by wealth, education level, urban/rural area and indigenous ethnicity. Public Health Nutr. 2020;23:s77–88. 10.1017/S1368980019004725 32148210 PMC10201355

[19] European Observatory on Health Systems and Policies. Health system performance assessment: a framework for policy analysis. 1st ed. Papanicolas I, Rajan D, Karanikolos M, Soucat A, Figueras J, editors. Copenhagen, Denmark: World health organization; 2022. 37023239

[20] Harris J Nisbett N The basic determinants of malnutrition: resources, structures, ideas and power. Int J Health Policy Manag. 2021;10:817–27. 33590741 10.34172/ijhpm.2020.259 PMC9309972

[21] Setyawan FEB Lestari R Holistic-comprehensive approaches to improve nutritional status of children under five years. J Public Health Res. 2021;10:1–6. 10.4081/jphr.2021.2183 33855400 PMC8129771

[22] Aman F Rauf A Ali R Hussain J Ahmed I Balancing complex signals for robust predictive modeling. Sensors (Basel). 2021;21:8465. 10.3390/s21248465 34960557 PMC8706336

[23] Haque MA Choudhury N Wahid BZ Ahmed SMT Farzana FD Ali M A predictive modelling approach to illustrate factors correlating with stunting among children aged 12–23 months: a cluster randomised pre-post study. BMJ Open. 2023;13:e067961. 10.1136/bmjopen-2022-067961 37185644 PMC10151845

[24] Morgenstern JD Rosella LC Costa AP de Souza RJ Anderson LN Perspective: big data and machine learning could help advance nutritional epidemiology. Adv Nutr. 2021;12:621–31. 10.1093/advances/nmaa183 33606879 PMC8166570

[25] Abdulla F Rahman A Hossain MM Prevalence and risk predictors of childhood stunting in Bangladesh. PLoS One. 2023;18:e0279901. 10.1371/journal.pone.0279901 36701381 PMC9879476

[26] Iqbal MS Palmer AC Waid J Rahman SMM Bulbul MMI Ahmed T Nutritional status among school-age children of Bangladeshi tea garden workers. Food Nutr Bull. 2020;41:424–9. 10.1177/0379572120965299 33084406

[27] Mansur M Afiaz A Hossain MS Sociodemographic risk factors of under-five stunting in Bangladesh: assessing the role of interactions using a machine learning method. PLoS One. 2021;16:e0256729. 10.1371/journal.pone.0256729 34464402 PMC8407547

[28] Harrison E Syed S Ehsan L Iqbal NT Sadiq K Umrani F Machine learning model demonstrates stunting at birth and systemic inflammatory biomarkers as predictors of subsequent infant growth – a four-year prospective study. BMC Pediatr. 2020;20:498. 10.1186/s12887-020-02392-3 33126871 PMC7597024

[29] Siy Van VT Antonio VA Siguin CP Gordoncillo NP Sescon JT Go CC Predicting undernutrition among elementary schoolchildren in the Philippines using machine learning algorithms. Nutrition. 2022;96:111571. 10.1016/j.nut.2021.111571 35063809

[30] Ndagijimana S Kabano IH Masabo E Ntaganda JM Prediction of stunting among under-5 children in Rwanda using machine learning techniques. J Prev Med Public Health. 2023;56:41–9. 10.3961/jpmph.22.388 36746421 PMC9925281

[31] Kirk D Kok E Tufano M Tekinerdogan B Feskens EJM Camps G Machine learning in nutrition research. Adv Nutr. 2022;13:2573–89. 10.1093/advances/nmac103 36166846 PMC9776646

[32] Salinari A Machì M Armas Diaz Y Cianciosi D Qi Z Yang B The application of digital technologies and artificial intelligence in healthcare: an overview on nutrition assessment. Diseases. 2023;11:1–12. 10.3390/diseases11030097 37489449 PMC10366918

[33] Romero-Martínez M Shamah-Levy T Franco-Núñez A Villalpando S Cuevas-Nasu L Gutiérrez J National health and nutrition survey 2012: design and coverage. Salud Publica Mex. 2013;55:S332–40. 24626712

[34] Romero-Martínez M Shamah-Levy T Vielma-Orozco E Heredia-Hernández O Mojica-Cuevas J Cuevas-Nasu L National health and nutrition survey 2018-19: methodology and perspectives. Salud Publica Mex. 2019;61:917–23. 31869555 10.21149/11095

[35] World Health Organization (WHO) Multicentre Growth Reference Study Group. WHO child growth standards: length/height-for-age, weight-for-age, weight-for-length, weight-for-height and body mass index-for-age: Methods and development. Geneva: World Health Organization; 2006.

[36] World Health Organization (WHO). WHO expert committee on physical status: the use and interpretation of anthropometry. WHO technical report series 845. Geneva: World Health Organization; 1995. 8594834

[37] Cuevas-Nasu L Gaona-Pineda EB Rodríguez-Ramírez S Morales-Ruán M del C González-Castell LD García-Feregrino R Stunting in children population in localities under 100 000 inhabitants in Mexico. Salud Publica Mex. 2019;61:833–40. 10.21149/10642 31869547

[38] Campos AP Vilar-Compte M Hawkins SS Association between breastfeeding and child stunting in Mexico. Ann Glob Health. 2020;86:145. 10.5334/aogh.2836 33262934 PMC7678557

[39] Walker SP Wachs TD Grantham-McGregor S Black MM Nelson CA Huffman SL Inequality in early childhood: risk and protective factors for early child development. Lancet. 2011;378:1325–38. 10.1016/S0140-6736(11)60555-2 21944375

[40] Instituto Nacional de los Pueblos Indígenas (INPI). Población indígena en hogares según pueblo indígena por entidad federativa, 2020. Población autoadscrita indígena y afromexicana e indígena en hogares con base en el Censo de Población y Vivienda 2020. 2022. Available: https://www.inpi.gob.mx/indicadores2020/. Accessed: 8 July 2023. Spanish.

[41] Instituto Nacional de los Pueblos Indígenas (INPI). Indicadores de la población indígena. Sistema de información e indicadores sobre la población indígena de México. Mexico City; 2015 Available: https://www.gob.mx/inpi/documentos/indicadores-de-la-poblacion-indigena. Accessed: 1 May 2020. Spanish.

[42] Yaschine I. Progresa-Oportunidades-PROSPERA, veinte años de historia. In: Hernández Licona G, De la Garza Navarrete TP, Zamudio Chávez J, Yaschine Arroyo I, editors. El Progresa-Oportunidades-Prospera: a 20 años de su creación. 1st ed. CONEVAL: Mexico City, Mexico; 2019. pp. 31–65. Available: https://www.coneval.org.mx/Evaluacion/IEPSM/Documents/Libro_POP_20.pdf. Accessed: 18 September 2021. Spanish.

[43] Frenk J Gómez-Dantés O Quasi-experimental study designs series—paper 3: systematic generation of evidence through public policy evaluation. J Clin Epidemiol. 2017;89:17–20. 10.1016/j.jclinepi.2017.03.013 28365310

[44] Parker S Todd P Conditional cash transfers: the case of Progresa/Oportunidades. J Econ Lit. 2017;55:866–915. 10.1257/jel.20151233

[45] McKenzie DJ Measuring inequality with asset indicators. J Popul Econ. 2005;18:229–60. 10.1007/s00148-005-0224-7

[46] Filmer D Pritchett LH Estimating wealth effects without expenditure data-or tears: an application to educational enrollments in states of India. Demography. 2001;38:115–32. 11227840 10.1353/dem.2001.0003

[47] Rencher A. Methods of multivariate analysis. 2nd ed. New York, NY, USA: John Wiley & Sons Inc; 2003.

[48] Poirier MJP Grépin KA Grignon M Approaches and alternatives to the wealth index to measure socioeconomic status using survey data: a critical interpretive synthesis. Soc Indic Res. 2020;148:1–46. 10.1007/s11205-019-02187-9

[49] Vyas S Kumaranayake L Constructing socio-economic status indices: how to use principal components analysis. Health Policy Plan. 2006;21:459–68. 10.1093/heapol/czl029 17030551

[50] Serván-Mori E Fuentes-Rivera E Quezada AD Pineda-Antunez C del Carmen Hernández-Chávez M García-Martínez A Early neurological development and nutritional status in Mexican socially deprived contexts. PLoS One. 2022;17:e0270085. 10.1371/journal.pone.0270085 35727758 PMC9212134

[51] Celhay P Martinez S Vidal C Measuring socioeconomic gaps in nutrition and early child development in Bolivia. Int J Equity Health. 2020;19:122. 10.1186/s12939-020-01197-1 32690012 PMC7370503

[52] Mohsena M Mascie-Taylor CGN Goto R Association between socio-economic status and childhood undernutrition in Bangladesh; a comparison of possession score and poverty index. Public Health Nutr. 2010;13:1498–504. 10.1017/S1368980010001758 20576197

[53] Li S Mohamed Nor N Kaliappan SR Social determinants of child malnutrition outcomes: Evidence from CHNS in China. Heliyon. 2023;10:e23887. 10.1016/j.heliyon.2023.e23887 38187311 PMC10767191

[54] Bommer C Vollmer S Subramanian SV How socioeconomic status moderates the stunting-age relationship in low-income and middle-income countries. BMJ Glob Health. 2019;4:e001175. 10.1136/bmjgh-2018-001175 30899561 PMC6407538

[55] Consejo Nacional de Población (CONAPO). Índice de marginación por entidad federativa y municipio 2020. Nota técnico-metodológica. Ciudad de México, México; 2021. Available: https://www.gob.mx/cms/uploads/attachment/file/685354/Nota_te_cnica_IMEyM_2020.pdf. Accessed: 15 January 2023. Spanish.

[56] Hastie T, Tibshirani R, Friedman J. The elements of statistical learning: data mining, inference, and prediction. 2nd ed. New York, NY, USA: SpringerLink; 2009.

[57] Kuhn M, Wickham H. Tidymodels: a collection of packages for modeling and machine learning using tidyverse principles. 2020. Available: https://www.tidymodels.org. Accessed: 15 January 2023.

[58] Chawla NV Bowyer KW Hall LO Kegelmeyer WP SMOTE: synthetic minority over-sampling technique. J Artif Intell Res. 2002;16:321–57. 10.1613/jair.953

[59] Kuhn M, Johnson K. Applied Predictive Modeling. 1st ed. New York, NY, USA: SpringerLink; 2013.

[60] Yu M Tham Y-C Rim TH Ting DSW Wong TY Cheng C-Y Reporting on deep learning algorithms in health care. Lancet Digit Health. 2019;1:e328–9. 10.1016/S2589-7500(19)30132-3 33323206

[61] Zheng Y Cheon H Katz CM Using machine learning methods to develop a short tree-based adaptive classification test: case study with a high-dimensional item pool and imbalanced data. Appl Psychol Meas. 2020;44:499–514. 10.1177/0146621620931198 34565931 PMC7495791

[62] Théodore FL Bonvecchio Arenas A García-Guerra A García IB Alvarado R Rawlinson CJ Sociocultural influences on poor nutrition and program utilization of Mexico’s conditional cash transfer program. J Nutr. 2019;149:2290S–301S. 10.1093/jn/nxz181 31793644 PMC13169019

[63] Fenta HM Zewotir T Muluneh EK A machine learning classifier approach for identifying the determinants of under-five child undernutrition in Ethiopian administrative zones. BMC Med Inform Decis Mak. 2021;21:291. 10.1186/s12911-021-01652-1 34689769 PMC8542294

[64] Shen H Zhao H Jiang Y Machine learning algorithms for predicting stunting among under-five children in Papua New Guinea. Children (Basel). 2023;10:1638. 10.3390/children10101638 37892302 PMC10605317

[65] Thomson S Achievement at school and socioeconomic background—an educational perspective. NPJ Sci Learn. 2018;3:5. 10.1038/s41539-018-0022-0 30631466 PMC6220286

[66] Gertler PJ, Boyce S. An experiment in incentive-based welfare: the impact of PROGRESA on health in Mexico. Annual Conference 2003 Royal Economic Society. 2003. Available: https://ideas.repec.org/p/ecj/ac2003/85.html. Accessed: 15 January 2023.

[67] Rukiko MD Mwakalobo ABS Mmasa JJ The impact of conditional cash transfer program on stunting in under five year’s poor children. Public Health Pract (Oxf). 2023;6:100437. 10.1016/j.puhip.2023.100437 37920185 PMC10618750

[68] Goudet SM Bogin BA Madise NJ Griffiths PL Nutritional interventions for preventing stunting in children (birth to 59 months) living in urban slums in low- and middle-income countries (LMIC). Cochrane Database Syst Rev. 2019;6:CD011695. 10.1002/14651858.CD011695.pub2 31204795 PMC6572871

[69] Tangcharoensathien V Mills A Das MB Patcharanarumol W Buntan M Johns J Addressing the health of vulnerable populations: social inclusion and universal health coverage. J Glob Health. 2018;8:020304. 10.7189/jogh.08.020304 30410733 PMC6204007

[70] Brierley S Lowande K Potter RA Toral G Bureaucratic politics: blind spots and opportunities in political science. Annu Rev Polit Sci. 2023;26:271–90. 10.1146/annurev-polisci-061621-084933

[71] Aguilera Vasquez N Daher J Do nutrition and cash-based interventions and policies aimed at reducing stunting have an impact on economic development of low-and-middle-income countries? A systematic review. BMC Public Health. 2019;19:1419. 10.1186/s12889-019-7677-1 31666032 PMC6820910

[72] Rafique S Afzal S Prevalence and predictors of stunting in children under five years of age. J Coll Physicians Surg Pak. 2023;33:449–56. 10.29271/jcpsp.2023.04.449 37190720

[73] Muche A Gezie LD Baraki AG Amsalu ET Predictors of stunting among children age 6–59 months in Ethiopia using bayesian multi-level analysis. Sci Rep. 2021;11:3759. 10.1038/s41598-021-82755-7 33580097 PMC7881183

[74] Nomura K Bhandari AKC Matsumoto-Takahashi ELA Takahashi O Risk factors associated with stunting among children under five in Timor-Leste. Ann Glob Health. 2023;89:63. 10.5334/aogh.4199 37780840 PMC10540702

[75] Grimmer J Roberts ME Stewart BM Machine learning for social science: an agnostic approach. Annu Rev Polit Sci. 2021;24:395–419. 10.1146/annurev-polisci-053119-015921

[76] Wiemken TL Kelley RR Machine learning in epidemiology and health outcomes research. Annu Rev Public Health. 2020;41:21–36. 10.1146/annurev-publhealth-040119-094437 31577910

[77] Leroy JL Ruel M Verhofstadt E The impact of conditional cash transfer programmes on child nutrition: a review of evidence using a programme theory framework. J Dev Effect. 2009;1:103–29. 10.1080/19439340902924043

[78] García-Guerra A Neufeld LM Bonvecchio Arenas A Fernández-Gaxiola AC Mejía-Rodríguez F García-Feregrino R Closing the nutrition impact gap using program impact pathway analyses to inform the need for program modifications in Mexico’s conditional cash transfer program. J Nutr. 2019;149:2281S–9S. 10.1093/jn/nxz169 31793648 PMC6887996

[79] Cuevas-Nasu L García-Guerra A González-Castell LD Morales-Ruan MDC Méndez-Gómez Humarán I Gaona-Pineda EB Magnitud y tendencia de la desnutrición y factores asociados con baja talla en niños menores de cinco años en México, Ensanut 2018-19. Salud Publica Mex. 2021;63:339–49. 10.21149/12193 34098606

[80] Cuevas-Nasu L Shamah-Levy T Arcos MA Gómez-Acosta LM Stunting Distribution in Mexico, An Unsolved Problem. Curr Dev Nutr. 2020;4:1391. 10.1093/cdn/nzaa061_019

[81] Cuevas-Nasu L Shamah-Levy T Hernández-Cordero SL González-Castell LD Méndez Gómez-Humarán I Ávila-Arcos MA Tendencias de la mala nutrición en menores de cinco años en México, 1988-2016: análisis de cinco encuestas nacionales. Salud Publica Mex. 2018;60:283–90. Spanish. 10.21149/8846 29746745

[82] Leyva-Flores R Serván-Mori E Infante-Xibille C Pelcastre-Villafuerte BE Gonzalez T Primary Health Care Utilization by the Mexican Indigenous Population: The Role of the Seguro Popular in Socially Inequitable Contexts. PLoS One. 2014;9:e102781. 10.1371/journal.pone.0102781 25099399 PMC4123888

[83] Serván-Mori E Meneses-Navarro S Garcia-Diaz R Flamand L Gómez-Dantés O Lozano R Inequitable financial protection in health for indigenous populations: the Mexican case. J Racial Ethn Health Disparities. 2024;11:3139–49. 10.1007/s40615-023-01770-8 37697143

[84] Gatica-Domínguez G Mesenburg MA Barros AJD Victora CG Ethnic inequalities in child stunting and feeding practices: results from surveys in thirteen countries from Latin America. Int J Equity Health. 2020;19:53. 10.1186/s12939-020-01165-9 32272935 PMC7147069

[85] Beech BM Ford C Thorpe RJ Bruce MA Norris KC Poverty, racism, and the public health crisis in America. Front Public Health. 2021;9:699049. 10.3389/fpubh.2021.699049 34552904 PMC8450438

[86] Medina M Barreto P Natero V Moratorio X Severi C Prevalence of malnutrition among children and women of reproductive age in Uruguay by socio-economic status and educational level. Public Health Nutr. 2020;23:s101–7. 10.1017/S1368980020000804 32299530 PMC10200402

[87] Zapata ME Soruco AI Carmuega E Malnutrition in all its forms and socio-economic indicators in Argentina. Public Health Nutr. 2020;23:s13–20. 10.1017/S1368980019003124 31685076 PMC8056984

[88] Curi-Quinto K Ortiz-Panozo E López de Romaña D Malnutrition in all its forms and socio-economic disparities in children under 5 years of age and women of reproductive age in Peru. Public Health Nutr. 2020;23:s89–100. 10.1017/S136898001900315X 31791443 PMC10200630

[89] Mazariegos M Kroker-Lobos MF Ramírez-Zea M Socio-economic and ethnic disparities of malnutrition in all its forms in Guatemala. Public Health Nutr. 2020;23:s68–76. 10.1017/S1368980019002738 31588883 PMC10200633

[90] Rajula HS Verlato G Manchia M Antonucci N Fanos V Comparison of conventional statistical methods with machine learning in medicine: diagnosis, drug development, and treatment. Medicina (Kaunas). 2020;56:455. 10.3390/medicina56090455 32911665 PMC7560135

[91] Bennett M, Hayes K, Kleczyk EJ, Mehta R. Similarities and differences between machine learning and traditional advanced statistical modeling in healthcare analytics. arXiv:2201.02469v1 [preprint]. 2022. Available: https://arxiv.org/abs/2201.02469. Accessed: 15 January 2023.

[92] Zhang A Xing L Zou J Wu JC Shifting machine learning for healthcare from development to deployment and from models to data. Nat Biomed Eng. 2022;6:1330–45. 10.1038/s41551-022-00898-y 35788685 PMC12063568

[93] Fernald LC Neufeld LM Overweight with concurrent stunting in very young children from rural Mexico: prevalence and associated factors. Eur J Clin Nutr. 2007;61:623–32. 10.1038/sj.ejcn.1602558 17136036

[94] Chunara R Gjonaj J Immaculate E Wanga I Alaro J Scott-Sheldon LAJ Social determinants of health: the need for data science methods and capacity. Lancet Digit Health. 2024;6:e235–7. 10.1016/S2589-7500(24)00022-0 38519151 PMC11001304

